# Ecotoxicity of Concretes with Granulated Slag from Gray Iron Pilot Production as Filler

**DOI:** 10.3390/ma10050505

**Published:** 2017-05-06

**Authors:** Helena Hybská, Emília Hroncová, Juraj Ladomerský, Karol Balco, Jozef Mitterpach

**Affiliations:** 1 Department of Environmental Engineering, Faculty of Ecology and Environmental Sciences, Technical University in Zvolen, Zvolen 960 01, Slovakia; hybska@tuzvo.sk (H.H.); jozef.mitterpach@gmail.com (J.M.); 2 Department of Environmental Management, Faculty of Natural Sciences, Matej Bel University, Banská Bystrica 974 01, Slovakia; juraj.ladomersky@umb.sk; 3 ZLH Plus a.s., Hronec 976 45, Slovakia; karolbalco@pobox.sk

**Keywords:** red mud, gray iron, granulated slag, concrete composite, ecotoxicity tests

## Abstract

This paper focuses on research concerning the ecotoxicological properties of granulated slag from the pilot production of gray iron with red mud addition and concrete composites with the application of this slag. Red mud is a hazardous waste generated in the production of aluminium oxide. Negative ecotoxicological tests are, therefore, one of the basic prerequisites for the ability to use granulated slag from gray iron pilot production. Granulated slag and concrete composite samples with various ratios of granulated slag have been subject to ecotoxicity tests: determining root growth inhibition in the highly-cultivated plant *Sinapis alba*, and determining acute toxicity in *Daphnia magna*. The results of ecotoxicological testing of granulated slag from gray iron standard production and gray iron pilot production with the additive were, according to the standard (STN 83 8303), negative. Additionally, the results of ecotoxicological tests of concrete composites were negative, with the exception of a 50% substitution of fine aggregate with slag from gray iron pilot production.

## 1. Introduction

Despite efforts to minimize waste generation and the use of the best available technique (BAT) technologies, anthropogenic activity still causes a large amonut of industrial waste. Industry is the source, in particular of inorganic wastes which, taking into account their amount and environmental quality, affect the overall negative environmental impacts of industrial production. 

In metallurgic processes, when producing pig iron, steels, cast irons, and non-ferrous metals, solid waste—slag—is generated. Both its composition and properties depend on the raw input material features, on the specific process, temperatures, time of thermal treatment, and more. Thus, methods of slag recovery, treatment, or disposal are also substantially influenced by the above characteristics. If waste from the metallurgical process is chemically qualified as non-hazardous industrial waste, then it can be reused in a variety of products [1]. Otherwise, it must be proven that the application of hazardous waste into a new product will not have a negative impact on the environment throughout its life cycle (from cradle to grave). For similar purposes, new and inexpensive tests must be developed for assessing environmental quality [2].

The slags from ferrous and non-ferrous metal production represent, in a majority of cases, problematic waste due to the presence of a wide variety of metals. Only a few slag groups from ferrous metal production—slag from cast iron—do not usually contain problematic elements. Slags with these properties are not very attractive for commercial applications. According to the European Standard STN EN 206 [3], only ground granulated blast furnace slag can be used as a major additive in cement production. Cupola slag is, with its physico-chemical properties, usually close to inert waste. However, if the intent is to use foundry slag, it is necessary to respect the chemical composition variability of the slag. Impacts of various technological modes of cast iron production can translate in the properties of the generated slag. Cupola foundry slag has different hydraulic properties than blast furnace slag and this standard does not allow ground granulated cupola foundry slag to be used as a cement additive [4].

Questions regarding the possibility of using foundry slags are subject to long-term applied research from the view of industrial ecology—to minimize raw material sources, reach maximum energy efficiency, and to minimize negative impacts on the environment [5]. Ham and Boyle [6] analyzed groundwater samples collected from monitoring wells at seven ferrous foundry landfills where a mixture of foundry sand, foundry slag, and foundry dust was disposed. Concentrations of toxic elements were smaller than limits at the time of the study. In contrast to results from lysimeter tests performed on foundry slag by [7], it is evident that concentrations of Cd and Cr may be a problem in some applications. Furthermore, based on the analysis reported in Bin-Shafique et al. [8] concentrations of these elements in water from lysimeter tests on foundry slag are higher than from tests on other foundry wastes.

Based on slag properties, it may be considered that the first option for their use is in the construction sector. The construction industry has a strong potential to replace natural raw materials with industrial inorganic waste, e.g., using foundry granulated slag or blast-furnace slag as a partial substitute in cement [4,9,10]. A well-known substitute for cement is fly ash and ground blast furnace slag. Basic properties of the raw input materials and the properties of the resulting building materials shall be explored [11,12]. The waste must be tested separately with certain types of tests, depending on its intended use [13,14,15]. 

From the viewpoint of a potential wider application of foundry slag, research not only of the pozzolanic and related material properties [4] is important. From an environmental viewpoint, construction materials containing waste may not be harmful for the environment or human health. Based on chemical analyses only, it is impossible to establish the overall impact of elements contained in the material under review on the environment or human health. We, thus, consider it necessary to also research foundry slag ecotoxicity, which is the focus of the presented paper. Its aim is the ecotoxicity research of concrete composites with partial substitution of aggregate with granulated slag from gray iron pilot production. In the pilot production of gray iron, an additive (compacted red mud) was added to the cupola intake according to [16]. Red mud is a hazardous waste, generated in the production of aluminium oxide. We followed the impact of granulated slag and concrete composites on two trophic levels, simulating the real environment.

## 2. Materials and Methods

### 2.1. Preparation of Concrete Composites

**Figure 1:**
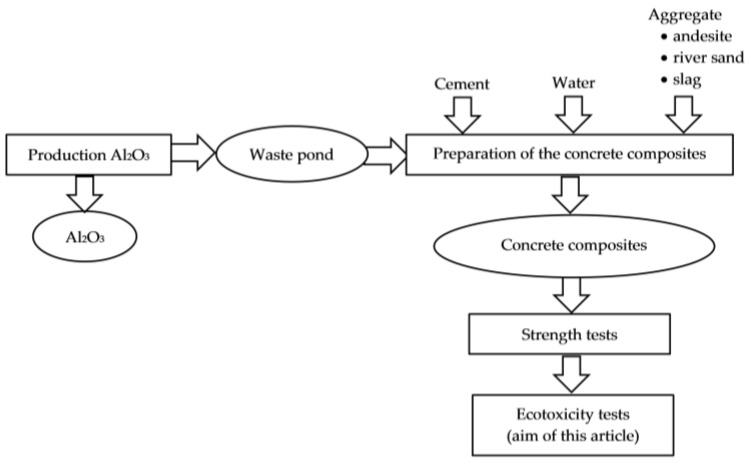
Waste flow and concrete composite preparation.

Concrete composites were prepared by mixing water, cement, sand, and fine aggregate, as per the formulation in Table 1. A part of the fine aggregate was substituted with granulated slag from gray iron pilot production in various ratios (Table 1). In the pilot production of gray iron, an additive was added to the intake (compacted red mud) with a ratio of 17 kg of the additive added per 1 tonne of intake [16]. Figure 1 shows the possibility of hazardous waste recovery of red mud generated in aluminium oxide production in gray iron pilot production in a cupola furnace, up to the subsequent recovery of the other waste, granulated slag in the production of concrete composites [17]. 

### 2.2. Sample Treatment and Water Leachate Preparation

**Figure 2:**
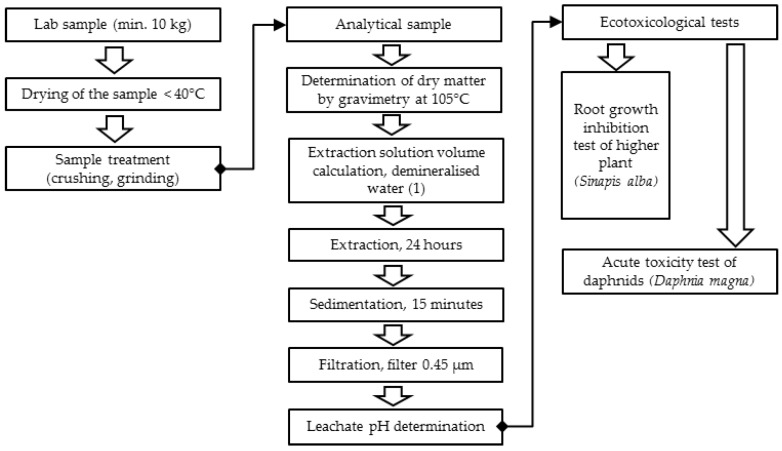
The treatment process of the concrete composites sample from the strength tests and the water leachate preparation [19].

For the purpose of the sample treatment, water leachate preparation, and ecotoxicity, methods were used that are acknowledged and recommended by competent international bodies [18]. The entire process of the concrete composite sample treatment from the strength tests and the water leachate preparation is schematically shown in Figure 2. The treatment of the concrete composite samples from the strength tests to a fraction under 4 mm and the leachate preparation were performed according to STN EN 14735 [19].

The volume of the extraction solution was calculated according to STN EN 14735 [19]:

L = (10 − M_C_/100) × M_D_, (1)

where L is the volume of the used extraction solution in litres, M_D_ is the weight of the dried tested part in kg, and M_C_ is the moisture content in percent. 

### 2.3. Methods to Determine the Physico-Chemical Properties of Leachates

pH Determination: The pH in samples of water leachates was determined potentiometrically with InoLab Terminal level 3 pH meter (WTW, Weilheim, Germany) with the use of SenTix 41 pH electrodes (WTW, Weilheim, Germany).

Determination of dissolved oxygen: The dissolved oxygen was determined by the electro-chemical method, using an OXI 340i oximeter with a StirrOX G membrane probe (WTW, Weilheim, Germany) after calibration.

Temperature determination: Measuring the temperature of samples was performed with a glass thermometer with a scale of measurement from 0–50 °C and divisions of 0.1 °C. 

### 2.4. Methods for Acute Ecotoxicity Determination

The ecotoxicity was tested in two types of granulated slag (from standard production and from pilot production) and five concrete composites (Table 1). Leaching tests were performed under STN EN 14735 [19] (Figure 2). The concrete composite samples collected after the strength tests were crushed and ground in a PM 400 planetary ball mill (Retsch, Germany) so that the resulting fraction contained particles of less than 4 mm. The extraction in demineralized water was performed by mixing it in a shaker ”head-heel” (Heidolph Reach 200, Heidolph Instruments GmbH & Co., Schwabach, Germany) at 10 revolutions per min and at a lab temperature of 22 °C. After sedimentation, membrane filtration was performed at a pressure of 60 kPa.

In order to determine the ecotoxicological properties, two tests according to STN 83 8303 were used [20]: the test of root growth inhibition in the highly-cultivated plant *Sinapis alba* and the acute toxicity test in *Dafnia magna*. 

The software STATISTICA 10 (Version 10, StatSoft, Tulsa, OK, USA), for ANOVA (analysis of variance) and single factor dispersion analysis, was used to evaluate the results of the ecotoxicological tests. The graphical presentation of ANOVA and Duncan’s test results were performed using 95% confidence intervals for average immobilization and inhibition values for individual samples.

#### 2.4.1. Root Growth Inhibition Test of the Highly-Cultivated Plant *Sinapis alba*

The impact of prepared water leachates on the highly-cultivated plant *Sinapis alba* root growth in the initial development phases was tested. The test consisted of cultivating seeds on mats saturated with water leachates prepared from the researched substance in comparison to seeds growing on a mat saturated with reconstituted water (control). thirty seeds of *Sinapis alba* were used for the test, and they were put on filtration paper in a Petri dish, and humidified with 10 mL of leachate. The test was performed in six repetitions. After 72 h, lengths of the roots were measured, based on which the root growth inhibition of *Sinapis alba* (Equation (2)) was calculated. The conditions are shown in Table 2.

The reconstituted water was prepared from the solutions, as shown in Table 3, namely by pipetting 10 mL of each of solutions 1–4, up to the volume of 1 litre, and was used as a control.

Inhibition, Li, of plant root growth shall be calculated according to Equation (2):

L_i_ = ((L_k_ − L_v_)/L_k_) × 100 (2)

where L_v_ is the average root length in the tested concentration of water leachate in cm; and L_k_ is the average root length in the control in cm.

The aim of the test under standard STN 83 8303 was to determine the acute toxicity of water leachate prepared from the samples of granulated slag and from the testing concrete composites [20]. In individual samples, a preliminary test was carried out in leachates, which were used without dilution.

#### 2.4.2. Acute Toxicity Test of *Daphnia magna*

This test was based on monitoring the immobilization of *Daphnia magna* in various concentrations of the tested substances after 24 and 48 h of exposition with a photoperiod of 16 h of light and eight hours of dark, at a stable temperature and without aeration, in comparison to the control (standard STN 83 8303) [20]. The conditions are shown in Table 4. The preliminary test consisted of putting 20 individuals of *Daphnia magna* into leachate without dilution. A control was also performed where reconstituted water was used instead of water leachate. The test was performed in six repetitions. 

The reconstituted water used for the control was prepared by pipetting 2.5 mL from each of solutions 1–4 (Table 3) into the volumetric flask with a volume of 1 litre and adding demineralized water up to the mark. The pH of the reconstituted water was adjusted to 7.8 ± 1.2 and the concentration of the diluted O_2_ was >7 mg/L. 

The aim of the present test was to determine the ecotoxicity concentration (EC), i.e., the concentration that immobilizes the percent of *Daphnia magna* individuals used in the test after 48 h of the test duration in comparison to the control.

The test result according to STN 83 8303 is, as follows [20]:

1. negative, if the immobilization or mortality of *Daphnia magna* ≤ 10% compared to the control; it shall be stated in the protocol and no further testing shall be performed.
2. positive, if the immobilization or mortality of *Daphnia magna* ≥ 10% compared to the control; in such a case:
  - If the immobilization or mortality of *Daphnia magna* < 50% compared to the control, it shall be stated in the protocol and no further testing shall be performed; and
  - If the immobilization or mortality of *Daphnia magna* ≥ 50%, the EC_50_ value shall be determined, and the orientation and basic tests shall be performed.

## 3. Results and Discussion

From several potential options of granulated foundry slag recovery going solely to landfills in Slovakia, we see two perspective options based on our research: concrete product production and the improvement of heavy soil quality for soil not used for farming purposes. Furthermore, adding finely-ground granulated slag from cupola to the cement slink, under patent by Stroup et al. [21], is promising. The basic precondition for the use of granulated slag in concrete composites is that the concrete composite should comply with standard physical and chemical properties. In the application of granulated slag from cast iron production, it is necessary to establish such properties not only in the short-term (e.g., of the strength after 2 h and 24 h), but also for the long-term (a minimum of 1 year) after the production of the concrete composite [4]. 

Granulated slag from gray iron standard production as a partial substitute for natural raw material in the production of concrete composites meets the long-term physical and mechanical properties [4]. Granulated slag from gray iron pilot production, in which the waste from aluminium oxide production was used, is being verified in an analogous long-term test. In this case, neither positive standard physical nor chemical properties will be sufficient.

In the previous period we have carried out research focused on the recovery of two types of slags derived from production of gray cast iron: generated within the manufacturing of concrete products [22] are two types of slag:

- granular slag from standard production in a cupola furnace; and
- granular slag from pilot production in a cupola furnace slag with a modified batch through red mud.

Testing of concrete cube production and determination of their properties were carried out according to the STN EN 12390-2 and EN 196-2 standards [23,24]. The results show that replacing up to 20% of fine aggregate with a granular slag from standard production of gray cast iron in a cupola furnace, a noticeably higher compressive strength of concrete cube than the strength of the standard were achieved [22]. In the case of fine aggregate replacement by granulated slag from pilot production of gray cast iron in a cupola furnace with modified batch through red mud, a slightly lower compressive strength was achieved. In all variants—in the case of less than 50% substitution of fine aggregates with slag of pilot production—the required minimum value of compressive strength of 30 MPa was achieved.

In many cases, ecotoxicological properties of the waste are decisive based on the material recovery of the specific waste. This is why samples from gray iron standard production and gray iron pilot production were tested. The test results are shown in (Table 5).

Table 5 shows that the determined values in both cases are, according to the applicable standard STN 83 8303 [20], negative. Based on these results, it can be stated that, for concrete production, principally, granulated slag from gray iron pilot production would be usable, in which hazardous waste would be used in the intake of raw materials. This confirms that waste foundry sand can have a more significant negative impact on the environment than granulated foundry slags [25]. 

The changes in physico-chemical properties are shown in Table 6 and they suggest that conditions for the validity of the acute toxicity test in *Daphnia* were complied with. Acute toxicity tests with *Daphnia magna* respond to a large variety of chemicals with high sensitivity [26]. 

However, understanding or improving ecotoxicological properties of granulated slags generated in gray iron production may contribute to their better use in the construction sector. The results Arum and Mark [27] indicate the suitability of granulated cupola furnace slag for use in concrete for which reduced permeability is an essential performance requirement. Similarly, the achieved results of Sharma et al. [28] suggested that reasonably high strength concrete can be designed by substituting fine aggregates with 10% to 45% of foundry slag and a partial replacement of cement with 15% of Alccofine.

After the ecotoxicity tests of granulated slag from gray iron standard production and gray iron pilot production, concrete composites were produced, using those slags as a partial substitute of aggregates. Subsequently, the ecotoxicity of the concrete composites prepared according to the formulation in Table 1 was examined. 

The leachates were prepared according to STN EN 14 735 with demineralized water used as the extraction solution [19]. From the values stated in Table 7, it reveals that the leachates were alkaline. The leachate from the testing subject marked CC0 had the lowest pH value and contained no granulated slag, and its value was 10.42. The most alkaline was the leachate marked CC10, where there was the highest addition of granulated slag from pilot production. Obviously, alkalinity of red mud showed up in concrete composites, even though the alkalinity of the granulated slags themselves was not as significant. This question will require further research.

### 3.1. Results of Root Growth Inhibition Testign of the Highly-Cultivated Plant Sinapis alba

**Figure 3:**
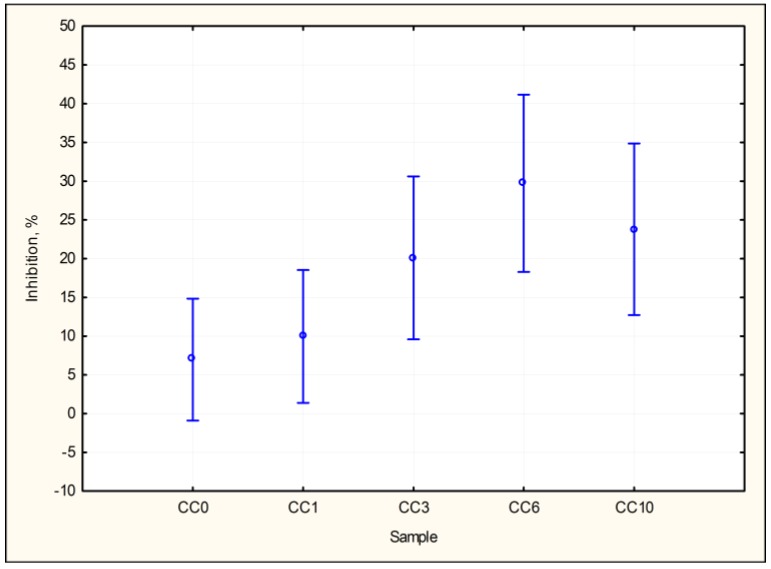
Graph of the 95% confidence intervals for inhibition by samples. Root growth inhibition test of *Sinapis alba*.

The results of the root growth inhibition test of *Sinapis alba* is shown in Figure 3 suggest a trend that, with increasing the ratio of granulated slag from gray iron pilot production in concrete composites, *Sinapis alba* root growth inhibition increases when compared to the control. The test was negative in all cases (root growth inhibition < 30%, or stimulation < 75% compared to control) and, therefore, it was not necessary to perform further testing (STN 83 8303) [20].

In order to correct the two-sample *t*-test, which, if used in more couples would produce incorrect results, the Duncan test was used. Based on the results of Table 8, there is a likelihood of conformity between CC0 and CC1 of 38.3%, between CC3 and CC10 of 28.6%, and between CC6 and CC10, there is 10.1% conformity.

The basic statistical characteristics of the analyzed water leachates of concrete composites tested with the root growth inhibition test of *Sinapis alba* are shown in Table 9.

### 3.2. Evaluation of Acute Toxicity Testing of Daphnia magna

**Figure 4:**
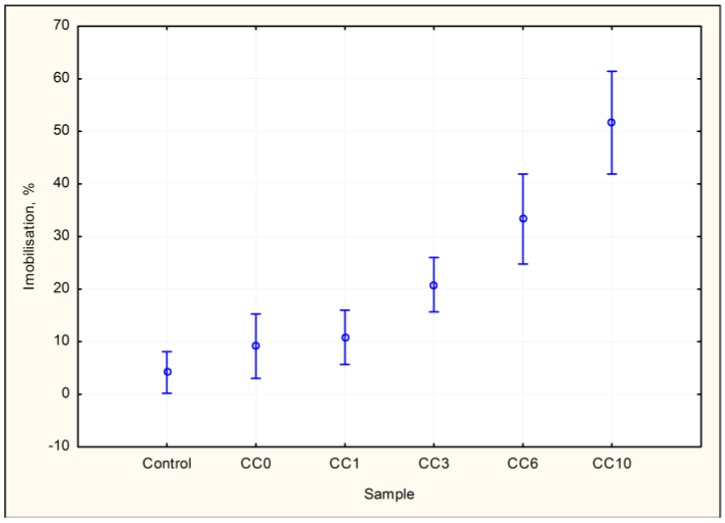
Graph 95% confidence intervals for imobilization by samples—acute toxicity test of *Dafnia magna.*

Taking into account the determined values (Figure 4) and conditions resulting from the preliminary test results, it was established that the results in samples, except the CC10 results, were negative (STN 83 8303) [20]. Additionally, in this case, there is a visible trend of increasing inhibition with a growing ratio of granulated slag in the concrete composite, as stated in Figure 3 of the root growth inhibition test of *Sinapis alba*.

The Duncan test (Table 10) shows a likelihood of conformity between the control and CC0 at 19%, between the control and CC1 at 10% and, for CC0–CC1, it is 65.8%.

For the basic statistical characteristics of the samples analyzed by way of the acute toxicity test of *Dafnia magna*, see Table 11.

The result of the test (Figure 4, Table 11) shows that the highest examined portion (50%) of aggregate substitution with slag from gray iron pilot production in the concrete composite, 51.7%, can already pose a risk.

In order to control the precision of the work according to GLP (Good Laboratory Praxis), a test with reference substance, potassium dichromate, was also performed, where the value (inhibition concentration) IC_50_ in the root growth inhibition test of *Sinapis alba* was 26.28 mg/L after 72 h and 0.62 mg/L after 48 h in the acute toxicity test of *Daphnia magna*. The determined values were at a 95% confidence interval and complied with the requirements of (STN 83 8303) [20]. 

Based on the achieved results, we can state that with increasing aggregate substitution in the concrete composites with foundry slag, the immobilization of individuals increases, thereby increasing the toxic impact of the samples, which, however, does not pose any potential toxic risk. 

Our analyses showed that ecotoxicological analyses have a decisive relevance in considering applications of waste-based products. Tests of the metal leaching limit of these products present additional information [29].

These results of ecotoxicological tests may support producers’ efforts to decrease the consumption of natural raw materials and replace them with wastes. At the same time, they provide evidence for the public that a waste-based product necessarily does not mean a risky one for man and nature. A thorough risk assessment (EPA, 2014) is always desirable [30].

## 4. Conclusions

The paper presents the results of ecotoxicity research in concrete composites that were produced with a partial substitution of up to 50% of fine aggregate (fraction 0/4 mm). As a partial substitute for fine aggregate, granulated slag from gray iron pilot production was used with the addition of coagulated red mud (in a ratio of 17 kg of coagulated red mud added per 1 tonne of intake) which is generated as hazardous waste in aluminium oxide production.

The ecotoxicity was tested in two types of slag (slag generated in standard and pilot productions) and five types of concrete composites with various substitutions of fine aggregate with slag from gray iron pilot production. Two tests of ecotoxicity were performed—*Sinapis alba* root growth inhibition testing and *Daphnia magna* acute toxicity testing (STN 83 8303) [20].

The results of ecotoxicological tests of slag generated from gray iron standard production and gray iron pilot production were negative. Additionally, the results of ecotoxicological tests of concrete composites were negative, with the exception of a 50% substitution of fine aggregate with slag from gray iron pilot production.

The ecotoxicity test results of concrete composites suggest a trend that with increasing the ratio of slag from gray iron pilot production in concrete composites, *Sinapis alba* root growth inhibition increases, compared to the control, as does the immobilization or mortality of *Daphnia magna*. 

The results of the ecotoxicity research confirmed that the application of the hazardous waste in the production of gray iron produced waste slag with no toxic properties. However, high values of pH > 10 in water leachates of concrete composites with the application of granulated slag from the pilot production were established. This aspect will require further research.

## Figures and Tables

**Table 1:** Formulations (percent by mass) of the preparation of concrete composites with the application of granulated slag from the pilot production of gray iron.

Sample	Cement ^1^ (%)	Water (%)	Coarse Aggregate (Andesite Fraction 8/16) ^2^ (%)	Fine Aggregate (River Sand Fraction 0/4) ^3^ (%)	Fine Aggregate (Slag Fraction 0/4) (%)
CC0	16.4	8.6	38.5	36.5	0
CC1	16.4	8.6	38.5	34.7	1.8
CC3	16.4	8.6	38.5	31.0	5.5
CC6	16.4	8.6	38.5	25.6	10.9
CC10	16.4	8.6	38.5	18.2	18.3

CC: cement composite; ^1^ Cement: CEM II/B-S 32.5 R (Cementáreň Ladce, a.s., Slovakia); ^2^ Badín, Slovakia; ^3^ Holiša, Slovakia.

**Table 2:** Test conditions of *Sinapis alba* growth inhibition.

Testing Organism	*Sinapis alba*, Ochre Yellow, Size 1.5–2 mm, Germination > 90%, 30 Seeds in a PETRI Dish, 10 mL Sample
Temperature	20 °C ± 1 °C, incubator TS 606 CZ/2-Var (WTW, Germany).
Control	Reconstituted water (Table 3)
Exposition period	72 h
Monitored response	Root growth inhibition compared to control, IC

**Table 3:** Stock solution for the preparation of reconstituted water.

Stock Solution	Chemical Substance	Concentration (g∙L^−1^)
1	CaCl_2_·2H_2_O, p.a.	117.6
2	MgSO_4_·7H_2_O, p.a.	49.3
3	NaHCO_3_, p.a.	25.9
4	KCL, p.a.	2.3

**Table 4:** Acute toxicity test conditions for *Daphnia magna*.

Testing Organism	*Daphnia magna*
Organism age	Individuals younger than 24 h since birth
Control	Reconstituted water (Table 3)
Sample volume	10 mL
Incubation temperature	20 °C ± 2 °C
pH	7.8 ± 0.2
Test duration	24 h/48 h
Validity criterion	Control: immobilization ≤ 10%, change in concentration of the diluted O_2_ ≤ 2 mg/L
Monitored response	% of immobilized individuals compared to the control, pH, temperature, diluted O_2_

**Table 5:** Ecotoxicological test results of granulated slag from gray iron production.

Biotest	Slag	
	GS1	GS2
Root growth inhibition test of *Sinapis alba*	29.10% inhibition	39.10% inhibition
Acute toxicity test of *Daphnia magna*	5% of immobilized individuals	0% of immobilized individuals

GS1: granulated slag from gray iron standard production; GS2: granulated slag from gray iron pilot production.

**Table 6:** Physico-chemical indicators of water leachates of slag over the acute toxicity test of *Daphnia magna.*

Sample	pH		Dissolved Oxygen [mg/L]		Temperature [°C]	
	0 h	48 h	0 h	48 h	0 h	48 h
Control	7.99	7.99	100.2	98.2	20.5	20.7
GS1	8.47	8.45	100.5	98.3	20.5	20.7
GS2	8.09	8.04	106.2	98.2	20.6	20.5

GS1: granulated slag from gray iron standard production; GS2: granulated slag from gray iron pilot production.

**Table 7:** Physico-chemical indicators of water leachates of concrete composites over the acute toxicity test of *Daphnia magna.*

Cement Composite	pH		Dissolved Oxygen [mg/L]		Temperature [°C]	
	0 h	48 h	0 h	48 h	0 h	48 h
Control	7.87	7.89	9.50	9.25	20.5	20.5
CC0	10.42	10.40	9.75	9.50	20.5	20.5
CC1	12.10	12.05	9.80	9.55	20.5	20.5
CC3	12.05	12.05	10.25	9.90	20.5	20.5
CC6	12.07	12.01	10.15	9.85	20.5	20.5
CC10	12.11	12.08	9.65	9.15	20.5	20.5

**Table 8:** Confidence levels for the Duncan test. *p*-value for the Duncan test for the root growth inhibition test of *Sinapis alba.*

Cement Composite	CC0	CC1	CC3	CC6	CC10
Average	6.97	9.97	20.10	29.73	23.80
CC0	-	0.383	0.003	0.000	0.001
CC1	0.383	-	0.012	0.000	0.002
CC3	0.003	0.012	-	0.019	0.286
CC6	0.000	0.000	0.019	-	0.101
CC10	0.001	0.002	0.286	0.101	-

**Table 9:** Basic statistical characteristics of test results of root growth inhibition of *Sinapis alba.*

Cement Composite	Inhibition (%)		95% Confidence Intervals		N
	Average	Standard error	Lower limit	Upper limit	
CC0	7.0	1.8	−0.9	14.8	6
CC1	10.0	2.0	1.4	18.5	6
CC3	20.1	2.4	9.6	30.6	6
CC6	29.7	2.7	18.3	41.2	6
CC10	23.8	2.6	12.7	34.9	6

N: number of repetitions

**Table 10:** Confidence levels for the Duncan test. *p*-value for the Duncan tes for the acute toxicity test of *Dafnia magna*.

Cement Composite	Control	CC0	CC1	CC3	CC6	CC10
Average	4.17	9.17	10.83	20.83	33.33	51.67
Control	-	0.190	0.100	0.000	0.000	0.000
CC0	0.190	-	0.658	0.005	0.000	0.000
CC1	0.100	0.658	-	0.012	0.000	0.000
CC3	0.000	0.005	0.012	-	0.002	0.000
CC6	0.000	0.000	0.000	0.002	-	0.000
CC10	0.000	0.000	0.000	0.000	0.000	-

**Table 11:** Basic statistical characteristics of acute toxicity test results of *Dafnia magna.*

Cement Composite	Inhibition (%)		95% Confidence Intervals		N
	Average	Standard Error	Lower Limit	Upper Limit	
Control	4.2	1.54	0.22	8.12	6
CC0	9.2	2.39	3.03	15.30	6
CC1	10.8	2.01	5.67	15.99	6
CC3	20.8	2.01	15.67	25.99	6
CC6	33.3	3.33	24.76	41.90	6
CC10	51.7	3.80	41.90	61.44	6

## References

[1] (2002). Beneficial Reuse of Foundry Sand: A Review of State Practices.

[2] Wieczerzak M., Namieśnik J., Kudłak B. (2016). Bioassays as one of the Green Chemistry tools for assessing environmental quality: A review. Environ. Int..

[3] (2015). Concrete. Specification, Performance, Production and Conformity.

[4] Ladomerský J., Janotka I., Hroncová E., Najdená I. (2016). One-year properties of concrete with partial substitution of natural aggregate by cupola foundry slag. J. Clean. Prod..

[5] Bourg D., Erkman S., Chirac J. (2003). Perspectives on Industrial Ecology.

[6] Ham R., Boyle W. (1990). Characteristics of Ferrous Foundry Wastes. Mod. Cast..

[7] Sauer J.J., Benson C.H., Edil T.B. (2005). Metals Leaching from Highway Test Sections Constructed with Industrial Byproducts.

[8] Bin-Shafique S., Benson C.H., Edil T.B. (2002). Leaching of Heavy Metals from Fly Ash Stabilized Soils used in Highway Pavements.

[9] Ceccato D.M., Masuero A.B., Moraes C.A.M., Vilela A.C.F. (2009). The recycling of Foundry granulated slag (FGS) as a partial substitute of cement in concrete. Revista Matéria.

[10] Safi B., Benmounah A., Saidi M. (2011). Rheology and zeta potential of cement pastes containing calcined silt and ground granulated blast-furnace slag. Mater. Constr..

[11] Eštokova A., Kovalčiková M., Luptaková A., Prascaková M. (2016). Testing silica fume-based concrete composites under chemical and microbiological sulfate attacks. Materials.

[12] Ondová M., Vaclavik V. (2015). Environmental Assessment of the Concrete Based on Blast Furnace Slag. Solid State Phenom..

[13] Harbuľáková V.O., Eštoková A., Luptáková A. (2016). Study of Dependencies between Concrete Deterioration Parameters of Fly Ash-Based Specimens. Dependability Engineering and Complex Systems.

[14] Junák J., Števulová N., Ondová M. (2014). Concrete samples prepared with different types of wastes. Pollack Period..

[15] Schneider N., Stephan D. (2016). Reactivation of a retarded suspension of ground granulated blast-furnace slag. Materials.

[16] Ladomerský J., Hroncová E., Nosáľ E., Matejka M., Jančovič P. (2012). The Additive from Waste Production of Alumina for Metallurgical Applications and Method for Obtain Thereof. Patent.

[17] Ladomerský J., Hroncová E. Two stage bauxite residue utilis ation method. Proceedings of the Bauxite Residue Valorisation and Best Practices.

[18] (2016). Series on Principles of Good Laboratory Practice and Compliance Monitoring.

[19] (2006). Characterization of Waste. Preparation of Waste Samples for Ecotoxicity Tests.

[20] (2015). Testing of Dangerous Properties of Wastes. Ecotoxicity. Acute Toxicity Tests on Aquatic Organisms and Growth Inhibition Tests of Algae and Higher Cultivated Plants.

[21] Stroup W.W., Stroup R.D., Fallin J.H. (2003). Cupola Slag Cement Mixture and Methods of Making and Using the Same. U.S. Patent.

[22] Hroncová E., Ladomerský J., Nosáľ E. (2011). Research of utilization of foundry sands and sands from forms and cores originated in aluminium casting production. Symposium Waste Forum 2011.

[23] (2010). Testing Hardened Concrete. Part 2: Making and Curing Specimens for Strength Tests.

[24] (2005). Methods of Testing Cement. Part 2: Chemical Analysis of Cement.

[25] Mastella M.A., Gislon E.S., Pelisser F., Ricken C., da Silva L., Angioletto E., Montedo O.R.K. (2014). Mechanical and toxicological evaluation of concrete artifacts containing waste foundry sand. Waste Manage..

[26] Martins J., Oliva Teles L., Vasconcelos V. (2007). Assays with Daphnia magna and Danio rerio as alert systems in aquatic toxicology. Environ. Inter..

[27] Arum C., Mark G.O. (2014). Partial Replacement of Portland Cement by Granulated Cupola Slag—Sustainable Option for Concrete of Low Permeability. Civil Environ. Res..

[28] Sharma D., Sharma S., Goyal A. (2016). Utilization of Waste Foundry Slag and Alccofine for Developing High Strength Concrete. Int. J. Electrochem. Sci..

[29] Yuvaraj M.T., Palanivel D.M., Vigneswar S., Bhoopathy R., Gnanasekaran V. (2015). Environmental Feasibility in Utilization of Foundry Solid Waste (Slag) for M20 Concrete Mix Proportions. IOSR J. Environ. Sci. Toxicol. Food Technol..

[30] (2014). Risk Assessment of Spent Foundry Sands in Soil-Related Applications: Evaluating Silica-Based Spent Foundry Sand from Iron, Steel and Aluminum Foundries.

